# Applications of Micro-Fourier Transform Infrared Spectroscopy (FTIR) in the Geological Sciences—A Review

**DOI:** 10.3390/ijms161226227

**Published:** 2015-12-18

**Authors:** Yanyan Chen, Caineng Zou, Maria Mastalerz, Suyun Hu, Carley Gasaway, Xiaowan Tao

**Affiliations:** 1 PetroChina Research Institute of Petroleum Exploration & Development, Beijing 100083, China; zcn@petrochina.com.cn (C.Z.); husy@petrochina.com.cn (S.H.); taoxiaowan@petrochina.com.cn (X.T.); 2 Indiana Geological Survey, Indiana University, Bloomington, IN 47405-2208, USA; mmastale@indiana.edu; 3 Department of Geological Sciences, Indiana University, Bloomington, IN 47405-1405, USA; cagasawa@umail.iu.edu

**Keywords:** micro-FTIR, coal, shale, fluid inclusion, chemical mapping, geological sciences

## Abstract

Fourier transform infrared spectroscopy (FTIR) can provide crucial information on the molecular structure of organic and inorganic components and has been used extensively for chemical characterization of geological samples in the past few decades. In this paper, recent applications of FTIR in the geological sciences are reviewed. Particularly, its use in the characterization of geochemistry and thermal maturation of organic matter in coal and shale is addressed. These investigations demonstrate that the employment of high-resolution micro-FTIR imaging enables visualization and mapping of the distributions of organic matter and minerals on a micrometer scale in geological samples, and promotes an advanced understanding of heterogeneity of organic rich coal and shale. Additionally, micro-FTIR is particularly suitable for *in situ*, non-destructive characterization of minute microfossils, small fluid and melt inclusions within crystals, and volatiles in glasses and minerals. This technique can also assist in the chemotaxonomic classification of macrofossils such as plant fossils. These features, barely accessible with other analytical techniques, may provide fundamental information on paleoclimate, depositional environment, and the evolution of geological (e.g., volcanic and magmatic) systems.

## 1. Introduction

Fourier transform infrared spectroscopy (FTIR) can provide fundamental information on the molecular structure of organic and inorganic components, and is one of the most versatile analytical techniques for the non-destructive, chemical characterization of geological samples, such as coal, shale, fluid and melt inclusions, silicate glass, minerals, and microfossils, e.g., [1,2,3,4,5,6]. The underlying mechanism of the FTIR technique is associated with transitions between quantized vibrational energy states [7]. In FTIR analysis, absorption of IR radiation occurs when a photon transfers to a molecule and excites it to a higher energy state [8]. The excited states result in the vibrations of molecular bonds (*i.e.*, stretching, bending, twisting, rocking, wagging, and out-of-plane deformation) occurring at varying wavenumbers (or frequencies) in the IR region of the light spectrum. The wavenumber of each IR absorbance peak is determined by the intrinsic physicochemical properties of the corresponding molecule, and is thus diagnostic, like a fingerprint of that particular functional group (e.g., C–H, O–H, C=O, *etc.*). A detailed introduction of the fundamental mechanism of this technique can be found in Griffiths and de Haseth [7] and Smith [9]. Molecules with dipole moments are IR detectable, and the majority of inorganic and organic compounds in the environment are IR active [7]. Much of the FTIR-related literature in geological sciences focuses on the mid-infrared (MIR) region of light (approximately 4000 to 400 cm^−1^).

The absorbances of molecular vibrations under IR radiation are proportional to the abundance of the functional groups. The absorbance of each vibrational band is often measured by the maximum height or the integrated area between the peak and a baseline. Several algorithms are available to define a baseline. The most widely used baseline is a linear line that is tangent to the minima on each side of the peak [3]. This type of baseline is easy to define and highly reproducible between operators [5]. Alternatively, baselines have been defined with a flexicurve or French curve [10,11], the combinations of Gaussians [12], and using spectra of analyzed samples of the same composition except that the molecules of interest are absent [13].

The concentration of the compound of interest can be determined from the IR absorbance using the *Beer-Lambert* *Law* (or simply *Beer’s Law*):

(1) $$A=-log_{10}\frac{I}{I_{0}}=\epsilon \cdot l\cdot c$$

where ***A*** is the absorbance (dimensionless), and *I* and *I_0_* denote the intensities of transmitted light and incident light. Parameters ϵ (in L·mol^−1^·cm^−1^), *l* (in cm), and *c* (in mol·cm^−1^) stand for the molar absorptivity, sample thickness, and molar concentration, respectively. The modified *Beer-Lambert* *Law* [14,15] is used more often in the geological sciences:

(2) $$w=\frac{A\cdot M}{\epsilon \cdot l\cdot \rho }$$

with *w* as the wt. % of the species of interest within the sample, *M* as the molar mass (in g·mol^−1^), and *ρ* as the density (in g·mol^−1^). Equation (2) requires the absorbance of the species, sample density, thickness, and the molar absorptivity of a sample to determine the concentration of the species in the sample. The modified *Beer’s Law* can be directly applied to samples containing simple components such as volatiles in glasses or inclusions which mainly consist of H and C species. However, proper mathematical algorithms are required for the multicomponent quantification of more complex samples like coal and shale, principally owing to the overlap of the characteristics peaks of many components. This issue will be elaborated in Section 2.3.

### 1.1. Conventional FTIR Techniques for Bulk Sample Characterization

**Figure 1:**
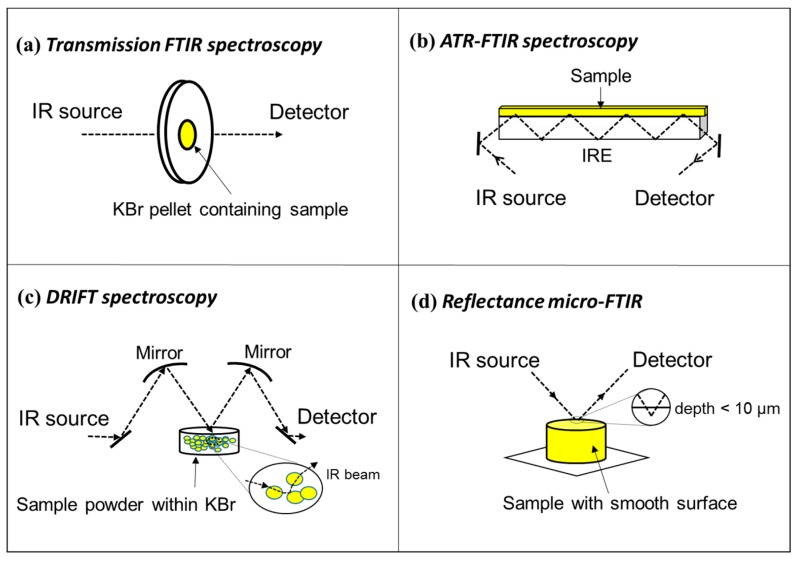
Simplified schematics of common Fourier transform infrared spectroscopy (FTIR) analysis modes including: (**a**) transmission FTIR; (**b**) attenuated total reflectance (ATR)-FTIR. Note that the penetration depth is dependent on the physical characteristics of internal reflection element (IRE) material and the angle of incidence [7]; (**c**) diffuse reflectance infrared Fourier transform (DRIFT) spectroscopy; (**d**) reflectance micro-FTIR. The penetration depth for reflectance micro-FTIR is usually less than 10 μm. Modified after figure 1 in Parikh and Chorover [8] with copyright permission from Taylor & Francis.

Most commonly used FTIR techniques for bulk sample analysis are transmission FTIR (e.g., potassium bromide (KBr)-pellet FTIR), attenuated total reflection (ATR)-FTIR, and diffuse reflection infrared Fourier Transform (DRIFT) spectroscopy (Figure 1a–c). Transmission FTIR is a fast and relatively cost-efficient technique which has been used extensively in chemistry, geology, and other scientific fields [1,2,16]. In this approach, the sample pellet is placed in the path of the IR beam and the resulting transmitted IR signal is recorded by the detector (Figure 1a). A KBr pellet is prepared by applying sufficiently high pressure to a homogenous mixture of KBr and the pulverized sample until the pellet turns transparent. KBr is used as the background matrix because it is IR transparent. The major challenge is to estimate the right proportion of the sample material in the pellet so that the resulting peak absorbances are not too weak nor too intense (preferably between 0.2 to 0.7 absorbance units). The linearity of *Beer*’s *Law* holds well when the absorbance is <0.7 [17]. Additionally, the analytical sample must be translucent enough (usually KBr pellets must be no more than 0.5–1 mm thick) to allow abundant light to pass through and reach the detector [8]. ATR-FTIR spectra provide chemical information on functional groups distributed near the surface of an internal reflection element [7,18,19]. Unlike transmission FTIR, IR radiation is not transmitted through the sample in ATR-FTIR, and consequently, the sample does not need to be prepared as a thin pellet. Moreover, the incorporation of the ATR crystal allows IR spectra with improved signal-to-noise ratios to be obtained with FTIR [18]. An additional advantage of ATR-FTIR is the relative ease of collecting quality data in the presence of water, which enables the examination of aqueous species sorption at crystal interfaces [20,21]. However, it is noteworthy that band intensities of ATR-FTIR spectra differ from those of transmission FTIR spectra owing to the interaction between IR beams and the ATR crystal [22]. This effect needs to be corrected by multiplying the spectrum with a linear function [18]. Diffuse reflection infrared Fourier Transform (DRIFT) spectroscopy requires simpler sample preparation compared with transmission FTIR. In DRIFT, the IR beam penetrates the analytical sample to a certain depth, and is then re-emitted from the sample and focused by a mirror onto the detector (Figure 1c). The resulting DRIFT spectrum is similar to that obtained by transmission FTIR technique [8], although the former is more dependent on physical characteristics of samples like absorptivity and reflectance [23]. DRIFT quantitative analysis requires the use of the Kubelka-Munk (KM) function, which provides a correlation between reflectance and sample concentration [24].

Although widely used in the determination of chemical structure in a variety of materials, these conventional FTIR techniques are limited to the investigation of bulk sample rather than individual components occurring at fine scales [6,25]. This characteristic largely restricts the extensive application of conventional FTIR techniques to homogeneous geological samples, since the results on bulk sample are weighted averages that fail to capture the wealth of information expressed by the heterogeneity at high spatial resolution [26].

### 1.2. Micro-FTIR for in Situ High Resolution Characterization

Since the development of mercury-cadmium telluride (MCT) detector technology, the micro-FTIR technique has become increasingly popular in characterizing samples that are too small to be chemically analyzed by conventional FTIR techniques. This modern technique can collect IR signals at high spatial resolution (the beam size can be as small as 5 µm), and has great potential for the characterization of compositionally complex samples. More importantly, micro-FTIR enables *in situ*, non-destructive analysis without the demand for sample purification and concentration, a process which is necessary in conventional FTIR analysis and very likely leads to specimen loss and alteration of chemical properties.

Micro-FTIR characterization can be conducted either in transmitted or reflected light mode. The principle of transmitted light mode is similar to that presented in Figure 1a. Transmission micro-FTIR can provide high-quality spectra and has often been used in coal structure characterization [27]. Nonetheless, sample preparation is critical to benefit from the potential advantages of transmission micro-FTIR. The analytical samples need to be thin enough to transmit light (Figure 1d), which is of great technical difficulty for some geological samples, e.g., coal and shale.

In reflectance micro-FTIR, IR radiation reflects from a polished and smooth solid sample surface, and the resulting signal is processed with an appropriate mathematical transformation (e.g., Kramers-Kronig transformation) to achieve similar spectra as transmission micro-FTIR spectra. The Kramers-Kronig transformation corrects for transflectance and shifts bands to the positions comparable to those in the KBr pellet spectra [28,29]. In reflectance micro-FTIR analysis, samples are usually prepared as thick blocks with well-polished, smooth surfaces, in the same manner as for the standard organic petrology techniques [30]. Although care is necessary, sample preparation in reflectance micro-FTIR is much easier and more time efficient compared with that for transmission micro-FTIR as only one side of the sample has to be prepared and the thickness of the sample is not critical. This easier sample preparation promotes the increasing use of reflectance micro-FTIR in geology and other sciences recently [26,31,32,33,34,35,36,37,38,39,40,41,42,43,44,45]. The beam size of micro-FTIR typically ranges from 20–100 µm when using a conventional IR source, and can be reduced down to 3–5 µm using a synchrotron radiation source [46].

The coupling of micro-FTIR and visible light microscopy opens the possibility of visualization and mapping of functional group abundances and molecular arrangements in samples across 2D regions [36,40] or even 3D cubes [39]. In micro-FTIR mapping mode, the infrared spectrum at each sampling point is measured and integrated, and parameters like peak areas in defined spectral regions are then used to map the distributions of target functional groups [46]. The acquisition time of micro-FTIR mapping depends largely on experimental settings, including the total number of sampling spots and scan numbers for each sampling spot, and may vary from hours to days. Recently, FTIR imaging performed with bi-dimensional arrays (e.g., focal plane array detectors, FPA) has become increasingly attractive for the speed at which data can be collected. In FPA imaging mode, the whole image is obtained in a single data collection by the use of multichannel detection, and a typical FPA-micro-FTIR image can be completed in a few minutes [46]. FPA’s fast data-collecting speed facilitates an even more exciting application, namely 3D FPA-micro-FTIR imaging. Operating like conventional X-ray CT microtomography, this approach rotates the sample through the IR beam and collects frequency attenuation images at different sample positions. The individual images are then processed to reconstruct a 3D rendering of the object, portraying the abundance and spatial distribution of IR-active chemicals within the sample [39].

In this paper, we will (i) present recent advances in the application of micro-FTIR techniques in a broad range of geological materials including natural and experimental glasses, minerals, inclusions and microfossils; and (ii) address in particular the chemical characterization of microscopic heterogeneity in coal and shale by using micro-FTIR and mapping techniques. The employment of reflectance-micro-FTIR mapping in geological samples, particularly shale, is still scarce in terms of fine-scale chemical characterization. This review underscores the great potential of non-destructive micro-FTIR technique in the high-resolution characterization of chemical heterogeneity in coal, shale, and other geological materials.

## 2. Application of FTIR to the Chemical Characterization of Coal and Shale

Organic matter (OM) contained in coal and shale is often heterogeneous, or comprised of numerous microscopically distinctive components. These components are usually on the micrometer scale and are referred to as macerals by coal and organic petrologists [47]. Chemically similar macerals can be generally grouped into the same type of kerogen, *i.e.*, macromolecular geopolymers which are technically defined as insoluble OM in commonly used organic solvents [48]. As a result of their microscopic sizes, the chemical characterization of individual macerals requires analytical tools that capture information at the micrometer scale to be used. This section will discuss the application of micro-FTIR for geochemical characterization and maturation of OM in coal (Section 2.1) and shale (Section 2.2), as well as FTIR quantification of organic and inorganic compositions in shale (Section 2.3).

### 2.1. Coal

**Figure 2:**
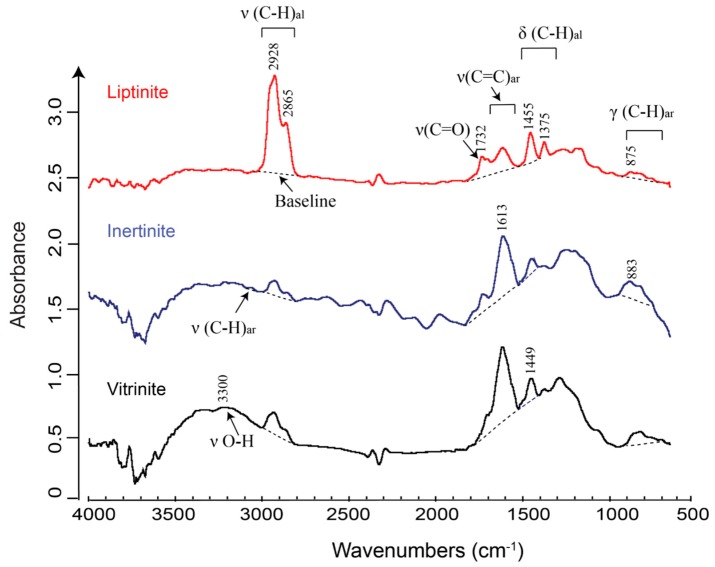
Micro-FTIR spectra of liptinite, vitrinite, and inertinite. Liptinite shows the strongest aliphatic CH_x_ stretching signal and the most intense oxygenated group stretching at ~1710 cm^−1^, but the lowest intensities for C=C ring stretching at ~1600 cm^−1^, aromatic CH_x_ stretching, and out-of-plane deformation. Dashed lines represent the linear baselines applied during the FTIR analysis. Note integrated areas of oxygenated and aromatic peak can be obtained by Fourier self-deconvolution [51] or curve-fitting of peaks in the ~1560–1800 cm^−1^ region [26]. Note υ—stretching vibration; δ—deformation vibration in plane; γ—deformation vibration out of plane; subscript al—aliphatic; subscript ar—aromatic. The spectra of liptinite and inertinite have been moved up the vertical axis to avoid overlaps. Modified after figure 6 in Chen *et al.* [26] with copyright permission from Elsevier.

Coal, a highly heterogeneous, organic-rich sedimentary rock, consists largely of helophytic and aquatic plant debris and derivatives ([49] and references therein). Coal macerals originating from various parental materials exhibit extraordinarily complex chemical properties, including a large variety and abundance of functional groups. Owing to the remarkably intricate chemical structure of coal, investigating coal with FTIR initially encountered great challenges, with major controversies being associated with the correct assignments of diagnostic bands to particular functional groups [50,51,52,53]. Recently, FTIR has received renewed interest among those studying coal following improvements in accessories and spectral processing techniques [18]. IR absorption frequencies of fundamental organic and inorganic functional groups in coal are listed in Table 1 [50,51,52,54,55], and typical micro-FTIR spectra of coal macerals are shown in Figure 2.

**Table 1:** Band assignments for the infrared spectra of coals [50,51,52,54,55].

Organic Functional Groups			Inorganic Functional Groups		
Wavenumber (cm^−1^)	Assignment	Ref	Wavenumber (cm^−1^)	Assignment	Ref
3300	OH stretch intermolecular bonding	[50,52]	3698, 3652,1095, 1034, 908, 689, 528, 338	Kaolinite, Al_2_(Si_2_O_5_)(OH)_4_	[52,54]
3010	Aromatic C–H	[50,51,52]	1464, 705, 245	Aragonite, CaCO_3_	[54]
2950	Aliphatic CH_3_	[50,51,52]	1450, 882, 729	Dolomite, CaMg(CO_3_)_2_	[54]
2920, 2850	Aliphatic CH, CH_2_, and CH_3_	[50,51,52]	1431, 869, 307	Calcite, CaCO_3_	[54]
1835	C=O, anhydride	[50]	3550, 3400,1615, 1155, 1132, 1106, 660	Gypsum, CaSO_4_·2H_2_O	[54]
1775–1765	C=O, ester with electron withdrawing group attached to single bonded oxygen	[50]	1115, 1148, 669	Anhydrite, CaSO_4_	[54]
1735	C=O ester	[50]	1075, 790, 452	Quartz, SiO_2_	[50,54]
1690–1720	C=O, ketone, aldehyde, and -COOH	[50]	1052	Smectite, A_0.3_D_2–3_[T_4_O_10_]Z_2_·nH_2_O ^¶^	[55]
1650–1630	C=O highly conjugated	[50]	1015	Oligoclase, (Na,Ca)[Al(Si,Al)Si_2_O_8_]	[55]
1600	Aromatic ring stretch	[50,51,52]	1006	Glauconite, (K,Na)(Mg,Fe)(Fe,Al)(Si,Al)_4_O_10_(OH)_2_	[55]
~1600	High conjugated hydrogen bounded C=O	[50]	1001	Muscovite, KAl_2_(AlSi_3_O_10_)(OH)_2_	[55]
1560–1590	Carboxyl group in salt from –COO^−^	[50]	984	Chlorite, A_5–6_T_4_Z_18_^§^	[55]
1490	Aromatic ring stretch	[50]	876, 727, 713	Ankerite, Ca(Fe,Mg)(CO_3_)_2_	[54]
1450	CH_2_ and CH_3_ bend, possibility of some aromatic ring modes	[50,51]	407, 396	Marcasite, FeS_2_	[54]
1375	CH_3_ groups	[50,52]	406, 340	Pyrite, FeS_2_	[54]
1300–1100	C-O stretch and O–H bend in phenoxy structures, ethers	[50]			
1100–1000	Aliphatic ethers, alcohols	[50]			
900–700	Aromatic C–H out-of-plane bending modes	[50]			
860	Isolated aromatic H	[50]			
833	1,4-substituted aromatic groups	[50]			
815	Isolated H and/or 2 neighboring H	[50]			
750	1,2-substituted, *i.e.*, 4 neighboring H	[50]			

^¶^: where A = Al, Fe, Li, Ca, Mg, or Ni, D = Al, Fe, Li, Mg, Cr, or Cu, T = Al, Si, and Z = O, OH. Data adopted from http://www.mindat.org/min-11119.html; ^§^: where A = Al, Fe, Li, Mg, Mn, or Ni, while T = Al, Fe, Si, and Z = O, OH. Data adopted from http://www.mindat.org/min-1016.html.

#### 2.1.1. Geochemistry of Coal Macerals Investigated by Micro-FTIR

Advanced insights into the geochemical properties of coal macerals are necessary for effective coal processing and utilization. Consequently, geochemical characterization of macerals has been the subject of numerous studies in recent years [18,19,26,32,33,34,35,37,38,40,56]. Purification and concentration by manual picking or physical separation is a crucial practice prior to collecting IR spectra when using conventional FTIR techniques on macerals [57]. Although useful information can be derived from the investigation of individual maceral concentrates, the physical separation process tends to cause specimen loss and introduce unexpected experimental errors [18]. In addition to the impossibility of obtaining clean concentrates, chemical properties of coal macerals can also be altered during the separation processes, especially when a strong acid or base is applied [37]. Moreover, the chemical and physical properties of macerals vary substantially, sometimes even within the same maceral group [32,33]. Because of this internal variability, bulk sample analysis often does not provide data that are insightful enough to understand coal utilization processes, and thus micro-techniques capable of high resolution measurements requiring minimal sample preparation have become indispensable for coal characterization.

Increasing attempts have been made to characterize individual coal macerals with micro-FTIR given its capability of portraying chemical information non-destructively at high spatial resolution. Commonly studied macerals are vitrinites, including macerals collinite and telinite [32,33,34,35,58], sporinite [59,60,61], resinite [37,38], cutinite [31,37,58], barkinite [41,60], alginite [31,58], fusinite and semifusinite [56], and funginite [38]. Although the coal samples may vary in ages and depositional environments, several common conclusions can be drawn from these investigations regarding functional group chemistry of liptinite, vitrinite, and inertinite maceral groups. Firstly, liptinite (e.g., sporinite, resinite, cutinite, alginite) contains the highest concentrations of aliphatic moieties and the lowest aromatic moieties compared to other maceral groups at similar maturities, whereas inertinite consists of the most aromatic and the least aliphatic moieties, with vitrinite displaying intermediate values for both proxies [26,37,38,58,61]. Secondly, vitrinite and inertinite have significantly lower CH_2_/CH_3_ ratios compared to liptinite, indicating that vitrinite and inertinite generally comprise shorter and more branched aliphatic chains containing higher terminal CH_3_ relative to methylene CH_2_ [31,62]. Finally, coal macerals are highly heterogeneous, and notable differences were observed even within the same particle of individual coal macerals [26,32,33,37,38]. This heterogeneity may be attributed to differences in depositional environments, digenetic processes, or botanical precursors [36].

**Figure 3:**
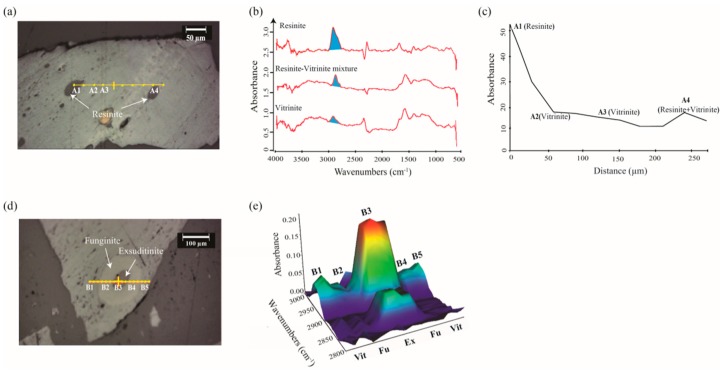
Chemical mapping of vitrinite, resinite, funginite, and exsudatinite in coal samples. (**a**) Photomicrograph of the sample in reflected light showing the location of transect ***A*** indicated by yellow dots. The sampling points are evenly distributed along the transect. The sampling area at point ***A1*** contains pure resinite, and points ***A2*** and ***A3*** represent pure vitrinite (Vit). The sampling area at point ***A4*** covers half resinite and half vitrinite; (**b**) FTIR spectra of resinite, vitrinite, and a resinite-vitrinite mixture. The blue-filled areas show how the absorbance plotted in panel (**c**) are defined. (**c**) the integrated area of aliphatic CH_x_ stretching at 3000–2800 cm^−1^ as a function of distance from point ***A1***. The aliphatic character is strongest in resinite (point ***A1***), decreases toward vitrinite (points ***A2*** and ***A3***) and increases in the approach to point ***A4*** that receives a mixed signal from both vitrinite and resinite; (**d**) Photomicrograph of the sample in reflected light showing the location of transect ***B*** (yellow dots) through a funginite (Fu) maceral; points ***B***1 and ***B***5 are in the adjacent vitrinite; points ***B2*** and ***B4*** are on the exterior funginite rim; point ***B3*** is on the exsudatinite (Ex) impregnation; (**e**) An alternative way of showing the variation in FTIR-spectral absorbance of aliphatic CH_x_ stretching bands at 3000–2800 cm^−1^ across transect ***B*** (shown in **d**) through funginite into adjacent vitrinite. Units in panels **c** and **e** are arbitrary absorbance units (AU). Modified after figures 4 and 8 in Chen *et al.* [38] with copyright permission from John Wiley and Sons.

Recent studies on vitrinite [26,59], sporinite, resinite, and funginite [38], and barkinite [41] have demonstrated the great potential for chemical imaging of coal macerals with the micro-FTIR mapping technique. Micro-FTIR mapping enables the visualization of chemistry of coal macerals across a certain microscopic region on a sample surface, and can be performed either in line or area modes. Line mapping contrasts the chemical properties along a certain intersection on a sample surface, and has been used to investigate coal samples containing vitrinite, resinite, exsudatinite, and funginite [38]. The intensity of aliphatic CH_x_ stretching has been observed to increase with a closer proximity to resinite (Figure 3c) and exsudatinite (Figure 3d), whereas it decreased towards funginite (Figure 3e).

**Figure 4:**
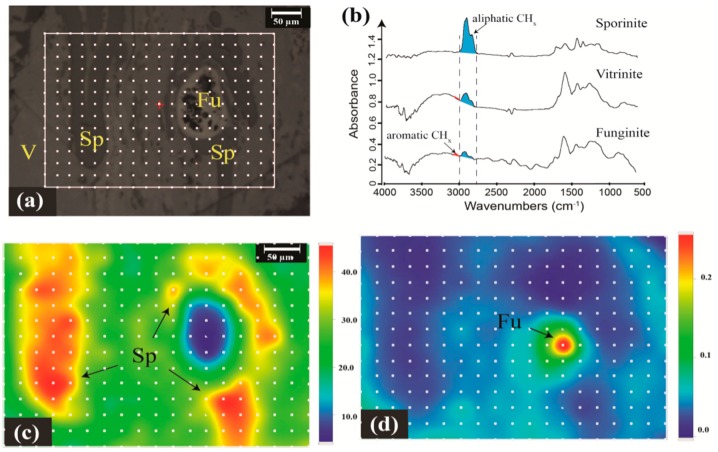
Chemical maps of a microscopic field with distinct sporinite (Sp), funginite (Fu), and vitrinite (V). (**a**) Photomicrograph in reflected light showing mapping sequence, indicated by yellow dots; (**b**) Representative FTIR spectra of sporinite, vitrinite, and funginite. The blue-filled areas represent the absorbance values that are mapped in panel (**c**); the ratios of red- over blue-filled areas are mapped in panel (**d**); (**c**) Chemical mapping of integrated peak of aliphatic CH_x_ groups (3000–2800 cm^−1^ region); (**d**) Map of the ratio of the integrated areas between 3100–3000 cm^−1^ and 3000–2800 cm^−1^ (*i.e.*, a proxy of aromaticity); Units in panels **c** and **d** are arbitrary absorbance units. Modified after figure 8 in Chen *et al.* [26] with copyright permission from Elsevier.

Area mapping is used more frequently in coal studies since it captures chemical properties and abundance of functional groups across the whole sampling region. A representative micro-FTIR mapping area study has been performed to investigate the chemical heterogeneity of a microscopic area featuring sporinite and funginite distributed among vitrinite, as is shown in Figure 4 ([26]). Micro-FTIR mapping displays the spectral absorbance of characteristic wavenumbers in color-coded images, in which red color denotes high signal intensity and blue color denotes low signal intensity. As demonstrated in Figure 4, sporinite features the most intense aliphatic CH_x_ peak at 3000–2800 cm^−1^ (red regions in Figure 4c), while funginite contains the strongest aromatic moieties (the red region in Figure 4d, showing the highest ratio between the 3100–3000 cm^−1^ peak and 3000–2800 cm^−1^ peak). Similar observations are made using micro-FTIR mapping and single-spot micro-FTIR analysis provided that micro-FTIR mapping is essentially an integration of the IR spectra of a series of single sampling spots.

More importantly, the non-destructive nature of micro-FTIR mapping enables the interactions between macerals to be observed *in situ*, which has previously eluded coal geologists [63,64]. The chemical mapping of a coal surface containing several resinite grains dispersed in vitrinite matrix indicated that aliphatic CH_x_ stretching intensity was higher for the vitrinite immediately adjacent to resinite than those more distant (figure 5 in Chen *et al.* [38]). This suggests that chemical components (such as aliphatic moieties) from resinite can diffuse into adjoining vitrinite. Contrary to this, funginite did not appear to influence the chemistry of adjacent vitrinite, which was attributed to the highly aromatic structure of this type of funginite [38].

#### 2.1.2. Maceral Coalification

Thermal maturation of coal (or coalification) advances as a result of increasing temperature and burial depth in sedimentary basins [65]. A variety of microbiological, chemical, and physical processes contribute to this intricate system. Detailed descriptions of the processes leading to coalification can be found in Stach [47]. Improved knowledge of these physicochemical processes and controlling mechanisms is of great significance for coal utilization and effective hydrocarbons-from-coal exploration and development.

**Figure 5:**
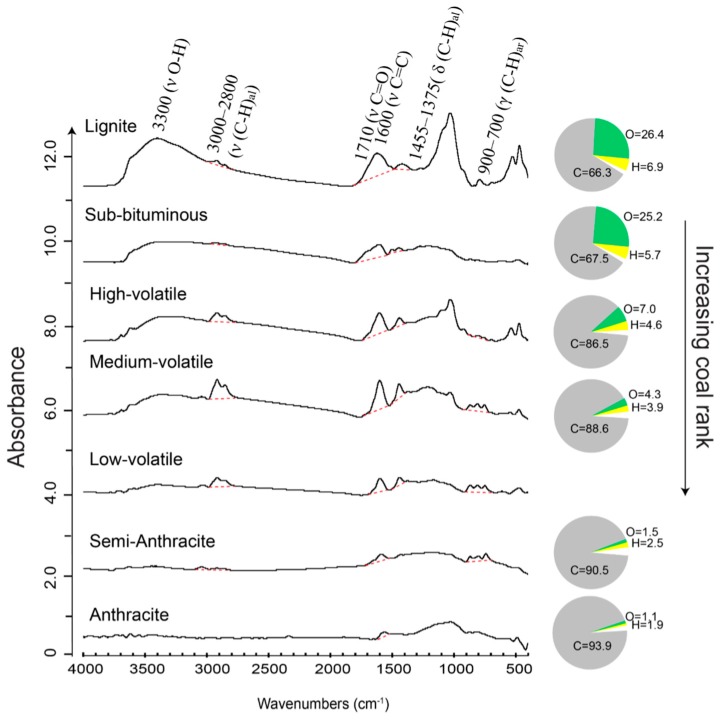
Micro-FTIR spectra of vitrinite in coals of different ranks. Adjacent pie diagrams represent elemental composition of these macerals measured by an electron microprobe. The hydrogen content is calculated by difference [33]. Red dashed lines represent the linear baselines applied to the FTIR spectra. Modified after figure 2 in Chen *et al.* [26] with copyright permission from Elsevier and Mastalerz and Bustin [33] with copyright permission from Elsevier. The elemental analysis results for low-volatile coal sample are not available.

Coal and coal macerals change their properties with increasing coalification; therefore determining the coalification level (termed coal rank) is of primary importance for coal scientists. Coal geologists document and compare the chemistry of coals with varying thermal maturities not only in order to better understand their complex maturation processes but also to make recommendations about their utilization. A large body of related work has been conducted in the past few decades on thermal maturities of coal macerals [26,28,33,40,66]. Coal samples with a wide range of coal ranks have been collected and analyzed by FTIR in combination with other independent methods, such as optical microscopy, electron microprobe, X-ray diffraction, SEM, *etc.* The microanalysis of macerals [26,28] (Figure 5) distinctly demonstrates that the abundance of functional groups and concentrations of elements like C, O, and H in coal macerals evolve in a consistent pattern in response to coalification. The percentage of C increases with thermal maturity, mainly in the form of condensed aromatic moieties, as reflected by the FTIR spectra. On the contrary, the IR absorbance of O/H-containing functional groups (e.g., CH_x_, O–H, C=O, *etc.*) drops with increasing maturation, which is in agreement with the decrease in the concentrations of H and O.

Artificially maturated coal samples or individual coal macerals have been used in some laboratories to trace the chemical evolution across increased thermal maturation gradients [53,67]. Most of these artificial maturation experiments are conducted in muffle ovens, or inert and ductile gold tubes, and the starting materials are commonly coal or macerals of low maturities. The thermal maturity of a coal sample increases as the heating temperature and dwell time increase. This way a series of samples with varying ranks can be synthesized. The major advantage of using artificially matured samples is that rock properties other than thermal maturity (e.g., mineralogical compositions, rock fabric, and grain contact) remain comparable among the whole suite of samples. Controversy still remains because short-term and intense artificial heating in a laboratory setting is very different from thermal maturation in nature, which is an extraordinarily long-term and gentle heating process [68]. Nevertheless, insightful and interesting results have been achieved with this approach in the past few decades. Mursito *et al.* [67] have observed a significant loss in O and an increase in C with thermal maturation, which caused a rise in carbon aromaticity and, thus, the sample’s hydrophobicity. Morga [56] has characterized a change in internal structure of semifusinite and fusinite in Upper Silesian coals (Poland) before and after heat-treatment (400–1200 °C). The results indicated that semifusinite and fusinite experienced an increase in aromaticity and condensation of the molecular structure, which was accompanied by a significant increase in reflectance under heating.

An alternative artificial practice can be conducted by coupling micro-FTIR with a diamond anvil compression cell (DAC). Although the credibility of the results can be partially hampered by the small sample size used that may be unrepresentative of the whole sample, micro-FTIR-DAC is capable of performing real-time monitoring of changes in chemistry in response to maturation, which makes the dynamic recording and analysis of geological processes possible. By using micro-FTIR-DAC, Ruau *et al.* [69] have been able to observe a progression of aromatization in vitrinite’s molecular structures through increasing coalification. In addition, they suggested that there was a functional rearrangement within the C=O groups during maturation. Micro-FTIR-DAC is particularly suitable for experiments requiring high temperature and pressure, which are unlikely to be achieved by a conventional artificial-maturation apparatus. When performed in mapping mode, micro-FTIR-DAC provides a new approach for the study of non-ambient chemical reaction dynamics, which we will describe in detail in Section 3.3.

Investigations by FTIR on OM transformations during thermal maturations have shown that with increasing thermal maturation: (i) OM expresses rising aromaticity and condensation of aromatic rings but decreasing aliphatic chain length [31,32,33]; (ii) the absorbance of oxygenated groups increases initially but decreases prominently later, which is likely the result of decarboxylation/dehydroxylation during coalification [47,66]; (iii) the ratio of CH_al_/(CH_al_ + C=C), a proxy of hydrocarbon-generating potential, rises in low-rank coal, but decreases in higher-rank coal [26]. These micro-FTIR studies and findings on coal improve our understanding of the fundamental processes occurring during maturation, as well as reaction mechanisms taking place during OM maturation and oil generation, which is of major importance in the development of models for petroleum exploration.

### 2.2. Shale

Shale is commonly composed of clay minerals, quartz, and fragments of other minerals (e.g., calcite and feldspar). It can contain variable amounts of OM. Shale has been attracting attention as worldwide shale gas and oil become more important resources. Shale rich in OM shares many petrologic and geochemical properties with coal. However, the characterization of shale is more challenging, because (i) multiple types of OM (primarily type I/II kerogen) are finely dispersed within the mineral matrix; and (ii) the coexistence of OM and various minerals creates highly heterogeneous shale compositions and severely complicates analyses.

**Figure 6:**
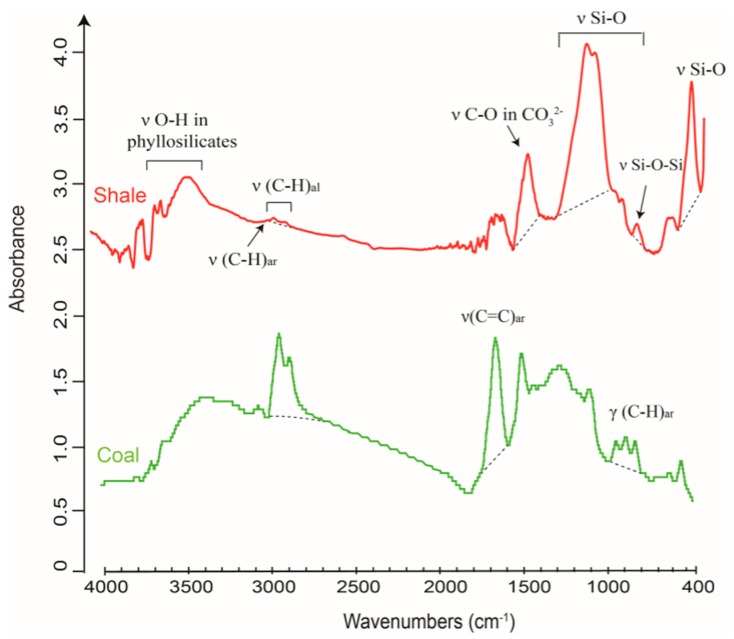
Comparison between the spectra of shale (red) and coal (green). Dashed lines are the linear baselines applied to the FTIR spectra. Note that the IR spectrum of shale contains strong absorbance of minerals occurring at 1500–400 cm^−1^, but low band intensity from organic matter at 3000–2800 cm^−1^.

Compared to the IR spectrum of coal, shale’s spectrum typically displays much weaker vibrational bands of aliphatic C–H at 3000–2800 cm^−1^, but features strong IR absorbance from minerals at 1500–400 cm^−1^ (Figure 6), which can mask aromatic and aliphatic peaks occurring in the same wavenumber range. The inorganic vibration bands may overlap the same group of minerals (e.g., calcite and dolomite) in the MIR spectral range, complicating FTIR qualification and quantification of mineral-abundant shale.

#### 2.2.1. Geochemistry of Organic Matter and Minerals in Shale

Prior to conventional FTIR analysis of OM in shale, pure kerogen has to be extracted and separated from bulk shale samples by repeatedly applying organic solvents, hydrochloric and hydrofluoric acids [70,71]. Micro-FTIR can be used directly on individual components of polished shale samples, including kerogen, removing the need to separate kerogen, greatly easing the sample preparation. In addition, kerogen-mineral interactions in shale can be documented *in situ* by micro-FTIR. Alstadt *et al.* [72] have studied the molecular properties of light and dark colored laminae of selected Green River oil shale (the Piceance Basin, CO, USA) with a step-scan photoacoustic FTIR. Their results revealed that mineral components significantly interacted with the kerogen molecules at molecular level, and the interaction acted differently between light and dark colored layers in the oil shale. The successful application of micro-FTIR to finely laminated shale also implies that this form of analysis will be useful in interpreting paleoclimate changes and cycles represented by differences in mineralogical composition of successive laminae over depth [73].

**Figure 7:**
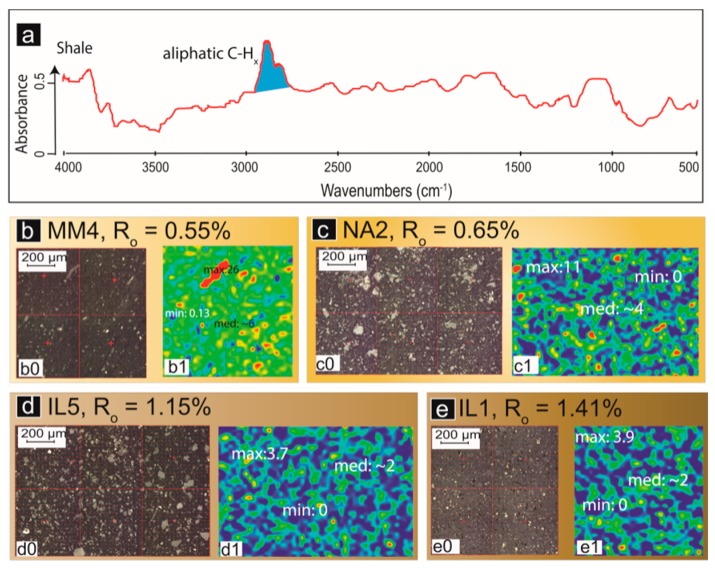
(**a**) A representative FTIR spectrum of the studied shale samples. The blue-filled areas represent the absorbance values that are mapped in panels ***b1***, ***c1***, ***d1*** and ***e1***. Micro-FTIR chemical maps of organic matter in (**b**) early mature shale MM4; ***b0***:photomicrograph; ***b1***:micro-FTIR chemistry map of integrated peak area of aliphatic CH_x_ groups; (**c**) mature shale NA2; ***c0***: photomicrograph; ***c1***:micro-FTIR chemistry map of integrated peak area of aliphatic CH_x_ groups; (**d**) late mature shale IL5; ***d0***: photomicrograph; ***d1***: micro-FTIR chemistry map of integrated peak area of aliphatic CH_x_ groups; and (**e**) postmature shale IL1; ***e0***: photomicrograph; ***e1***: micro-FTIR chemistry map of integrated peak area of aliphatic CH_x_ groups. Min, med, and max values stand for the minimum, median, and maximum values of integrated peak areas of aliphatic CH_x_ groups in 3000–2800 cm^−1^. Modified after figure 4 in Chen *et al.* [40] with copyright permission from John Wiley and Sons.

The hydrocarbon-generating potential of source rocks can be assessed by tracking physical and chemical changes in the molecular structure of kerogen through thermal maturation [17]. A routine approach to address this issue is the artificial maturation of immature, extracted kerogen, as described in Section 2.1.2. Micro-FTIR mapping of kerogen in shale provides a simplified approach to address this matter. Chemical maps of the New Albany Shale (Illinois Basin, IN, USA) samples of varying thermal maturities are displayed in Figure 7 (data from Chen *et al.* [40]). In response to increasing thermal stress, convertible carbon [74] gradually transforms into hydrocarbons that can later be expelled from the rock, and as a consequence the intensity of aliphatic groups decrease, as evidenced by the low intensity of aliphatic IR absorbance in highly mature samples (Figure 7d,e). Although largely controlled by total organic carbon (TOC) content and original OM arrangement, the abundance and spatial distribution of OM in shale can also be affected by OM migration and expulsion of hydrocarbons that shrink the volume of original kerogen [75,76].

**Figure 8:**
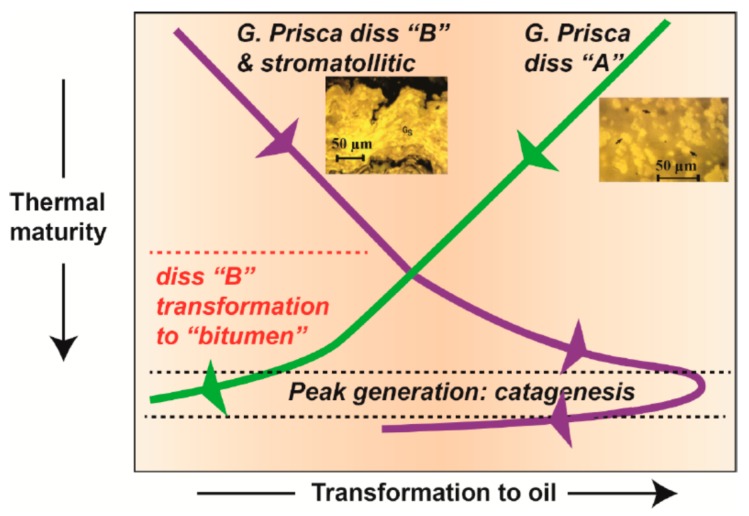
Model of petroleum generation for Yeoman Formation (Saskatchewan, Canada) *G. prisca* alginite [77]. Thin-walled *G. prisca* disseminated A contribute to pre-catagenic petroleum products. In contrast, thick-walled disseminated B alginite and stromatolitic *G. prisca* undergo a structural transformation into a bitumen-like maceral with increasing thermal maturity. Adjacent photomicrographs are those of *G. prisca* disseminated A and stromatolitic *G. prisca* alginite. Modified after figure 10 in Stasiuk *et al.* [77] with copyright permission from Elsevier.

Micro-FTIR has also proved to be useful in paleoenvironment reconstruction and hydrocarbon-generation model development. Transmission micro-FTIR studies on *G. prisca* alginite varieties (Upper Ordovician Yeoman Formation, Saskatchewan, Canada) indicated that thick-walled alginite varieties (disseminated B) and stromatolitic *G. prisca* contained stronger OH and aromatic CH_x_ absorbances than thin-walled alginite varieties (disseminated A) at the same level of thermal maturity [77]. This may be attributed to the formation of phenolic-rich biopolymers in the cell walls in response to a fluctuation in oxygen or salinity levels in the paleoenvironment [77]. The subtle changes in alga cell wall composition and structure have in turn imparted a fundamental control on the onset and amount of petroleum generation in response to thermal maturation (Figure 8). Specifically, disseminated A generates hydrocarbons during diagenesis, which is supported by the reduction in aliphatic CH_x_ absorption of the alginites with increasing thermal maturity (Figure 8). In contrast, the disseminated B variety progressively transforms from an alkyl long chain structure into a more cyclic bitumen maceral before peak generation, evidenced by the increased aliphatics with maturity in combination with other semi-quantitative FTIR data [77].

#### 2.2.2. FTIR Spectral Data as a Proxy for Porosity and Permeability

Porosity and permeability are among the most significant evaluation criteria for shale reservoirs, and substantial efforts have recently been devoted to develop effective and fast approaches for their characterization and quantification [78,79]. Tools commonly used for the investigation of porosity and permeability include high-resolution microscopic techniques which directly view pore geometry and morphology at fine scales, and probe-based porosimetric techniques measuring the porosity and permeability indirectly. Chen *et al.* [40] have developed an approach to relate shale chemical maps obtained via micro-FTIR to shale porosity and permeability, based on the observations that porosity in OM typically accounts for a large portion of total porosity in shale [80,81]. Then better interconnectivity of porous organic domains must be accompanied by the increased interconnectivity of pores in OM, and thus the permeability of shale. Indeed, positive correlations between the interconnectivity of OM domains, porosity, and Swanson permeability were observed by Chen *et al.* [40], indicating that interconnectivity of OM domains might be a useful proxy for shale permeability. Micro-FTIR, accompanied by porosimetric techniques, can improve our understanding of porosity networks in shale [40].

### 2.3. Quantification of Organic Matter and Minerals in Coal and Shale

“In the case of nearly all branches of science a great advance was made when accurate quantitative methods were used instead of merely qualitative.” (Sorby, 1908 [82]).

Abundant publications have highlighted the importance of fast and accurate quantification of organic and mineralogical components in coal [83], shale [84,85,86,87] and other sedimentary rocks [55,88,89] using FTIR techniques. Quantitative information about the compositions of rock and other geological samples can be obtained with FTIR tools since OM and minerals display most of their characteristic vibration modes in the IR spectral region [55]. The critical importance of sample preparation has been emphasized in several previous studies on transmission-FTIR quantification [55,83,84]. The absorbance is strongly related to a mineral’s grain size [84]. A large mineral grain size (>2.5 µm) may induce the dispersion of the IR beam [55]. Careful grinding is therefore required for transmission FTIR quantification.

Appropriate algorithms are also important in assuring credible FTIR quantification results, particularly for compositionally complex samples like coal and shale. The determination of the composition of a multicomponent sample from its FTIR spectrum is complicated by the overlap of IR bands and the nearly identical FTIR spectra of many components [55,85,88]. The measured absorbance is thus the sum of the absorbances of all the components (not only the component of interest) in the sample, and as a result the *Beer’s Law* cannot be directly applied to coal and shale. Proper algorithms are required to enable multicomponent FTIR quantification. A common procedure for multicomponent quantification is so-called “sequential subtraction” [83,84,85,86,87,88]. A library of the spectra of mineral and OM standards is used to sequentially subtract from the spectra of the coal/shale. The subtraction coefficient, which results in the complete elimination of the typical peaks of the particular component being subtracted, provides a measure of the weight fraction of that component present in the coal/shale [84]. Evident double peaks at 3000–2800 cm^−1^ from aliphatic C–H stretching are usually used for the quantification of OM. Characteristic bands of mineral components can be found in Table 1. Although sequential subtraction is straightforward and was used in a large body of FTIR quantification studies, the accuracy of the results generated in this way largely relies on subjective decisions about the subtracted spectrum, and analytical errors can become critical when determining low concentrations of certain components [83,87]. Least squares (LS) curve fitting has recently become an alternative computational tool for FTIR quantification. These results are more reproducible and objective compared to manual subtraction [50,55,74,90,91,92]. For instance, the LS regression of the spectra of 49 synthetic mineral mixtures predicted an average absolute difference between the known and derived mineral concentration of ±2.6 wt. % [55]. It is important that all of the components existing in the sample should be included in the LS regression model, otherwise the calculation is invalid. This shortcoming limits the application of LS in very complicated mixtures with unknown components. The feasibility of partial least squares (PLS) regression to DRIFTS and ATR-FTIR quantification of mineral mixtures has also been investigated, and shows considerable potential for accurate and precise mineralogical analysis [93,94]. PLS regression can be used to quantify complicated samples. However, the calculations tend to be rather slow. In addition, PLS requires a large number of mixture spectra to yield precise results, usually 3–5 times as many mixtures as constituents [7].

A non-negative least squares (NNLS)-FTIR quantitative study of an artificial mixture of multiple natural minerals, and shale samples from the Western Sedimentary Basin (Alberta, Canada) was recently presented by Chen *et al.* [73]. Limited errors below ±4 wt. % compared to the known mineral composition suggest that NNLS-FTIR is capable of rapid and accurate quantification of mineral fractions, kerogen in synthetic mixtures, and kerogen in natural shale. Using the same strategy, the micro-FTIR technique was tested for its applicability of *in situ*, high-resolution quantification of shale composition. Measured IR spectra agree more closely with a model prediction derived from NNLS-micro-FTIR than with a model prediction derived from NNLS-bulk FTIR, partially because analyses conducted by micro-FTIR avoid the necessity to crush and grind samples. The capability of performing high-spatial resolution quantification renders micro-FTIR as a particularly suitable tool for the quantitative portrayal of heterogeneities in coal and shale at small scales [73].

### 2.4. Limitations of Micro-FTIR in Coal and Shale Studies

Micro-FTIR possesses a few advantages over conventional transmission FTIR methods: (i) it alleviates the difficulties associated with sample preparation by using bulk samples instead of isolated macerals; and (ii) it analyzes samples non-destructively with intact inter-maceral or OM-mineral associations. These valuable attributes make this technique especially attractive in the analysis of microscopic heterogeneities in coal and shale in relation to their mineralogy and OM types.

There are, however, some inherent limitations associated with the application of this technique to coal and shale studies. One of these limitations is that micro-FTIR cannot reliably identify components smaller than 15 micrometers owing to the relative large aperture size of micro-FTIR with a conventional light source [26]. An additional drawback on OM characterization is the difficulty in identifying OM in highly mature shale, because aliphatic moieties in immobile OM (*i.e.*, kerogen, pyrobitumen) are chemically depleted during thermal maturation. Therefore, alternative approaches, for example using imaging processing software, have to be employed to discriminate between highly mature OM and minerals occurring on the surface via their contrasting color and reflectance intensity [40]. Another constraint comes from the fact that micro-FTIR, with a conventional IR source, only maps the chemical properties across a 2D surface, which may pose challenges to the translation of 2D chemical data to real 3D reservoir attributes. Two options may solve this problem: (i) the employment of multi-channel FPA-micro-FTIR 3D mapping [39]; or (ii) the integration of multiple micro-FTIR maps performed on surfaces perpendicular to each other following a Cartesian coordinate system.

A few noteworthy limitations also affect micro-FTIR quantification: (i) the great difficulty in obtaining pure mineral standards and homogeneous organic macerals that have unadulterated FTIR spectra [73]; (ii) the quantification errors of minor concentrations of certain minerals (e.g., phosphates and pyrite); and (iii) the inability to compare and calibrate micro-FTIR quantitative results with other independent analyses, primarily because quantitative information on the micrometer scale cannot currently be assessed with other techniques.

## 3. Other Geological Application of Micro-FTIR

### 3.1. Application to the Analysis of Volatiles in Glasses and Minerals

Volatiles (primarily H_2_O and CO_2_) drive volcanic eruptions [95]. Dissolved volatiles can strongly affect the physical properties and geochemical signatures of silicate melts and magmatic rocks [96,97]. Therefore, the characterization of volatiles is critical in order to improve the understanding of volcanic and magmatic processes. Methods commonly used for volatile analysis in volcanic rocks and minerals include Karl Fischer titration [98], FTIR [99,100], Raman spectroscopy [101,102], secondary ion mass spectrometry (SIMS) [103], and electron backscatter methods [104,105]. Among these approaches, FTIR is relatively simple, highly sensitive, and can provide quantitative measurements of different volatile species at high spatial resolution [10,106]. The use of synchrotron radiation micro-FTIR permits a spatial resolution of about 3–5 µm with the detection limits at the ppm level [107].

Sample preparation is an important step in guaranteeing satisfactory measurements using transmission FTIR. A doubly-polished wafer with parallel sides has to be prepared for the analysis [5]. The thickness of the wafer needs to be estimated prior to sample preparation. Optimal results are achieved when the maximum absorbance in the spectral range being used for quantitative analysis does not exceed 0.7 [17]. For example, a graph, such as figure 5 in von Aulock *et al.* [5] for rhyolitic glasses, can be used to estimate the optimum thickness for a volcanic glass sample. Additionally, appropriate adhesive for mounting the sample should be carefully selected based on their specific properties. Sample preparation can be tedious and challenging for fractured samples, fragile samples, such as pumices, and very small samples, such as inclusions within crystals.

The contents of H_2_O and CO_2_ can be quantitatively determined from IR spectra using the modified *Beer’s Law* (Equation (2)), which requires absorbance, density, thickness, and the molar absorptivity. The peak heights at ~3550 cm^−1^ and ~1630 cm^−1^ are commonly used to calculate the concentrations of total H_2_O (H_2_O_t_ ) and molecular H_2_O (H_2_O_m_) [108], and the contents of CO_2_ and CO_3_^2−^ can be determined from the peak heights of the bands at ~2350 cm^−1^ and ~1430 cm^−1^ [109,110]. The choice of baseline needs to be carefully considered as it may introduce large errors in absorbance measurements. The molar absorptivity (ϵ) is chemical-composition specific, and should theoretically be determined for every sample. However, literature values (see Table 1 in von Aulock *et al.* [5]) for similar chemical compositions offer sufficient accuracy in most cases. The density of natural glasses that are thin and heterogeneous can be calculated using the models developed by Lange and Carmichael [111] and Ochs and Lange [112]. The thickness (*l*) measurements are challenging, especially for thin and fragile samples. The most common way to determine sample thickness is directly using micrometers or digital displacement gauges, however, these can damage the samples if not performed carefully. Tamic *et al.* [113], Wysoczanski and Tani [114], and Nichols and Wysoczanski [115] used a new strategy to indirectly determine thicknesses by counting the interference fringes across a region of the reflectance FTIR spectra. Other tools for thickness measurements include calibrated microscope stages and optical profilometers. A good introduction to the approaches for thickness measurements is presented in von Aulock *et al.* [5].

Great efforts have recently been dedicated to developing reflectance micro-FTIR to analyze volatiles [42,44,45,108]. This modern FTIR technique requires only one exposed and polished surface, easing sample preparation. Reflectance micro-FTIR provides a means to analyze volatile abundances in fragile samples (e.g., cracked, vesicular glasses) and unusually H-rich samples that are otherwise impossible to analyze or prepare for analysis with transmission micro-FTIR techniques [42,43]. In addition, different depths within a single sample can be analyzed using sequential polishing procedures [44]. The application of the reflectance micro-FTIR also avoids the difficulties associated with thickness measurements. Using reflectance micro-FTIR, King and Larsen [44] have successfully retrieved linear functions relating H_2_O*_t_* contents with the Kramas-Kronig absorbance at 3550 cm^−1^ for basaltic, andesitic, phonolitic, and rhyolitic glasses. ATR micro-FTIR is an alternative FTIR-based technique that requires samples to be polished on only one side. Lowenstern and Pitcher [108] have determined H_2_O contents in hydrous glasses using ATR micro-FTIR, and the derived concentration values were comparable with those obtained by transmission micro-FTIR. ATR micro-FTIR yields higher sensitivity for a given optical aperture size than reflectance micro-FTIR [5,108]. However, ATR micro-FTIR can damage fragile samples through direct contact of the ATR accessory with the sample’s surface [108].

An increasing amount of publications have demonstrated the capabilities of micro-FTIR techniques in the high-resolution characterization of volatiles in natural, e.g., [10,116,117] and experimental glasses, e.g., [97]. The distribution and diffusion of H_2_O in the samples can facilitate the identification of the exsolution and re-dissolution of H_2_O in the melt [96,114,116], and provide better constraints on volcanic temperatures and timescales [5,99]. H_2_O distribution profiles created using micro-FTIR also assist in the recording and quantification of bubble evolution in the melt [43,97,118], which is a fundamental research topic in volcanology. Such studies are particularly important in understanding magmatic processes, and constructing and developing eruption mechanisms [5].

Recent advances in FTIR techniques allow H_2_O to be analyzed in nominally anhydrous minerals (NAMs) [4,119]. NAMs can accommodate H_2_O in the form of protons in defects in the mineral structure [120]. The study of H_2_O in NAMs is vital for constraining the H budget of Earth’s mantle, past and present mantle geodynamics, and the origin of volatiles in terrestrial planets [96,120]. Micro-FTIR maps displaying volatile distributions across minerals are of great interest to Earth scientists, as they may improve our understanding of crystallization processes [119]. Della Ventura *et al.* [121] observed an interesting compositional zonation of CO_2_ and CO_3_^2−^ contents in vishnevite from Latium, Italy, which may be induced by a major change in the physical conditions of the environment, such as temperature, during the mineral crystallization. The crystallographic orientations of the hydrous species in NAMs can be acquired using polarized FTIR [4,122].

### 3.2. Application to the Characterization of Fluid and Melt Inclusions

Crystals may contain small impurities of variable shape, referred to as inclusions [123], which are frequently grouped by the proportion of vapor, liquid or solid phase present in them at room temperature. Droplets consisting of liquid/vapor/daughter crystals are referred to as fluid inclusions, and melt inclusions are parcels of glass/vesicles/daughter crystals that were trapped within crystals as they grow in the magma [46,124]. Inclusions are a record of the geological environment in which the melt and/or fluids were trapped, and are thus of continuing interest to geologists [107,108,109,112,114,124].

Advances in analytical techniques have created an evolution in scientific methods used to characterize inclusions. Initially, bulk extraction was used, which is destructive, time consuming, and limited to the larger inclusions [106]. Recently, non-destructive micro-Raman spectroscopy has been developed to measure H_2_O in inclusions, e.g., [101,125]. Nonetheless, this technique requires calibrations, which largely depend on sample composition and analytical parameters that are currently laboratory dependent [115]. In recent years there has been an increased availability of synchrotron radiation, which enables the application of synchrotron-based FTIR imaging techniques to related studies, e.g., [107,126]. To achieve a good chemistry image of melt and fluid inclusions with transmission micro-FTIR, the host crystal has to be doubly polished in a careful manner so that the inclusion is completely exposed on both sides of the crystal slice [44,107,127]. That way, the IR beam interacts solely with the inclusion. The occurrence of H_2_O was successfully FTIR-mapped in fluid and melt inclusions hosted in crystals from Stromboli (Sicily, Italy), the Alban Hills volcano (Rome, Italy), and Franklin Furnace (Franklin, NJ, USA) (see figure 5 in Della Ventura [46]). The same technique was applied to study the distribution of volatile CO_2_ and H_2_O in an olivine-hosted melt inclusion hosted in a rock erupted at Procida Island, Southern Italy (figure 6 in Mormone *et al.* [110]).

Reflectance micro-FTIR requires only one surface to be exposed and polished, which is particularly suitable for the analysis of small inclusions in fragile samples [44,103]. Yasuda [45] has developed a method to measure H_2_O contents of silicate glasses by reflectance micro-FTIR. The method was calibrated using 32 synthesized glasses ranging from basalt to rhyolite in composition and a range of H_2_O contents. To apply the technique to melt inclusions he developed a method to correct for contamination from the host crystal calculating the overlapping volume of the host crystal using the reflectance spectra between 800 and 1300 cm^−1^. H_2_O contents of melt inclusions have also been determined using ATR micro-FTIR [108], which provides higher signal-to-noise ratios than reflectance micro-FTIR, and is thus well suited for imaging of volatiles in very small melt inclusions (<15 µm).

In addition to investigating inorganic H_2_O and CO_2_ in inclusions, the measurement and imaging of CH_4_ and other hydrocarbons can also be performed by micro-FTIR [128,129]. Barrès *et al.* [130] initiated the micro-FTIR analysis of hydrocarbon-bearing inclusions. This study demonstrated that micro-FTIR can distinguish various individual hydrocarbon fluid inclusions within a single sample, and this can be used to study the crude chemical evolution of hydrocarbons during the geological history of the sample [130]. However, because of IR source constraints, the quality of spectra was only sufficient for fluid inclusions with diameters larger than 30 µm to be successfully analyzed [130]. Applying sophisticated synchrotron-radiation micro-FTIR, Bourdet *et al.* [131] was able to map compositional variability within hydrocarbon inclusions in quartz using a beam size of ~3 μm. IR spectra of individual inclusions (figure 8 in Bourdet *et al.* [131]) were used to quantify methane concentrations, CH_2_/CH_3_ ratios and H_2_O–Hydrocarbon ratios.

### 3.3. Application to High-Temperature Studies

**Figure 9:**
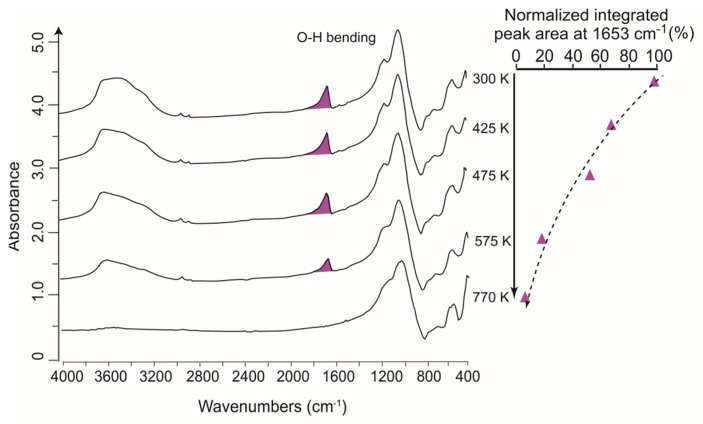
**Left**: Micro-IR spectra of stilbite collected at different temperatures (300, 425, 475, 575 and 770 K) in the 4000–400 cm^−1^ wavenumber range. Modified after figure 2 in Prasad *et al.* [134] with copyright permission from Mineralogical Society of America; **Right**: absorbance of H_2_O (represented by O–H bending, purple-filled area) with increasing temperature from 425 to 770 K normalized to that at 300 K. The normalized H_2_O absorbances were derived from corresponding FTIR spectra on the left and show dehydration with increasing temperature.

**Figure 10:**
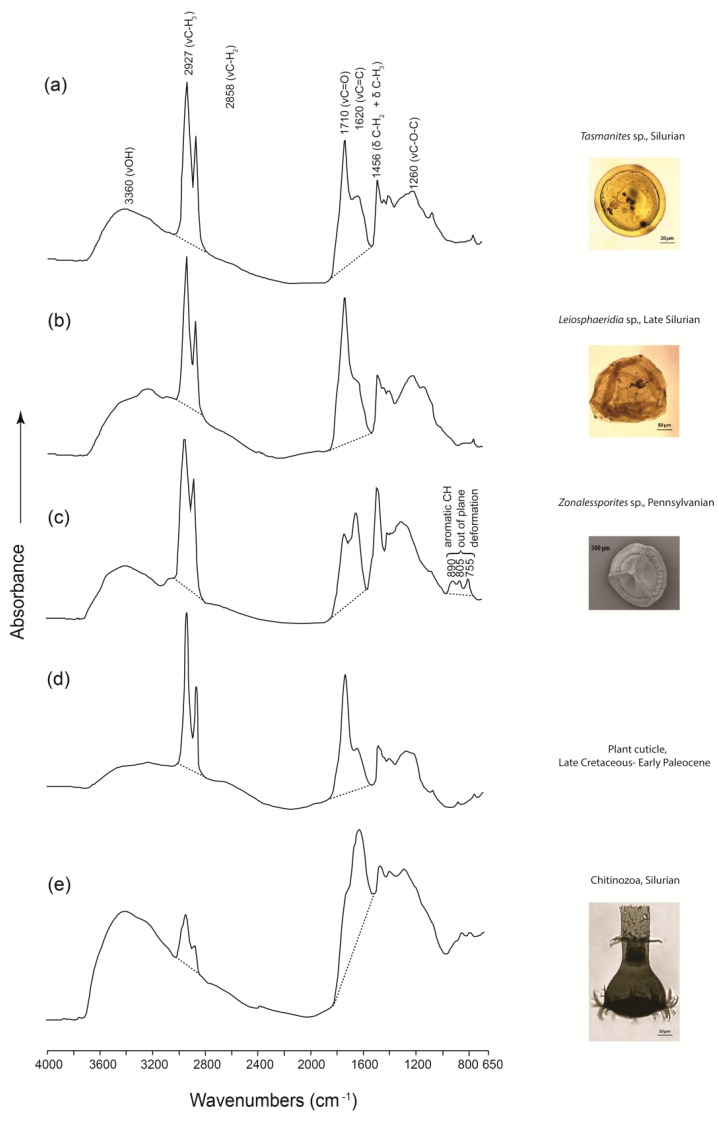
**Left**: Micro-FTIR spectra of prasinophycean algae (**a**) *Tasmanites* sp. from the Silurian, Oklahoma (USA) and (**b**) *Leiosphaeridia* sp. from the Late Silurian, Hazro area (Turkey); (**c**) a megaspore *Zonalessporites* sp. from the Pennsylvanian, Saarland (Germany); (**d**) a plant cuticle from the Late Cretaceous-Early Paleocene (western Germany); and (**e**) zoomorph Chitinozoa from the Silurian, Gotland (Sweden). Dashed lines indicate the linear baselines applied to the spectra. **Right**: Photomicrography of corresponding microfossils. Modified after figures 1–5 in Dutta *et al.* [139] with copyright permission from Elsevier.

FTIR, together with a diamond or sapphire cell and a heating stage (FTIR-DAC), has recently been employed in real-time monitoring of geochemical reactions taking place at high temperatures [132,133,134,135,136,137,138]. A few investigations have applied FTIR-DAC to processes in which the dehydration of H_2_O was involved [134,136,138]. In these studies FTIR spectra of the mineral sample were collected at a series of preset temperatures, and the extent to which the absorbance of a vibrational band associated with H_2_O (e.g., O–H peak) changes with temperature can be observed [134,136]. Dehydration ratios can be easily calculated by processing these temperature-dependent spectra, and plotted as a function of temperature to retrieve the kinetic information of mineral dehydration. For example, Prasad *et al.* [134] recorded the dehydration behavior of a natural stilbite sample from Poona (India) by employing transmission FTIR-DAC with the sample in a KBr pellet (Figure 9). Based on their FTIR spectra, the relative absorbance of H_2_O (*i.e.*, absorbance ratios of O–H band at an elevated temperature (*i.e.*, 425–770 K) over that at 300 K) was calculated and plotted *versus* temperature, as shown in Figure 10. The dehydration rate of stilbite can thus be easily derived from the tangential slops at various temperatures. Kinetic information on geochemical processes can also be achieved using micro-FTIR mapping/imaging. Della Ventura *et al.* [46] creatively applied micro-FTIR imaging to monitor the dehydration of a single crystal of leucite at high temperatures (see figure 6 in [46]). The images indicated that increasing temperature and time yielded clear dehydration.

### 3.4. Application to Microfossil Characterization and Chemotaxonomy

Increasing efforts have been made to employ micro-FTIR techniques to the study of microfossils, given the high sensitivity of this technique and the very low amount of specimen that it requires (typically 1–2 mg). Dutta *et al.* [139,140,141] used micro-FTIR to characterize well-preserved microfossils from Silurian and younger sedimentary sequences in the U.S., Turkey, Germany, and Sweden. The spectroscopic investigation indicated that the macromolecular structure of these microfossils consisted of both aliphatic and aromatic moieties (Figure 10). *Tasmanites, Leiosphaeridia*, and a plant cuticle had long-chain aliphatics and tended to be oil-prone, whereas the aliphatic chains in zoomorphs (*i.e.*, chitinozoans, scolecodonts, and arthropod cuticles) were short and more gas-prone in response to thermal stress. Megaspores exhibited moderate aliphatic and aromatic absorbance, and had both oil and gas generating potential [139].

**Figure 11:**
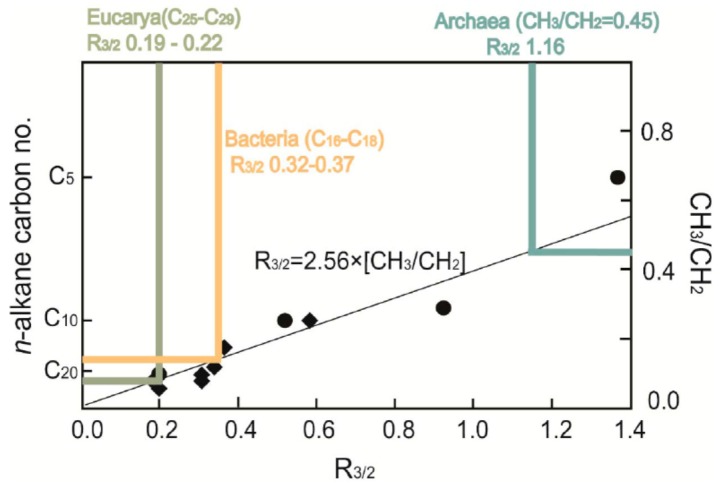
Relationship between R_3/2_ (aliphatic CH_3_/CH_2_ absorbance ratios) and ratio of CH_3_/CH_2_ for *n*-alkane standard samples (*n*-C_5_ to *n*-C_40_). The CH_3_/CH_2_ ratio can be converted to carbon number of the *n*-alkane. Modified after figure 6 in Igisu *et al.* [142] with copyright permission from Elsevier. Using this equation, R_3/2_ values of Eucarya, Bacterial and Archaeal lipids are estimated to be 0.19–0.22, 0.32–0.37, and 1.16, respectively [142]. The carbon number of Eucarya lipids (C_25_–C_29_) and bacterial lipids (C_16_–C_18_) are from Han and Calvin [143] and Ladygina *et al.* [144].

Microbial fossils with well-preserved morphological structures can provide direct evidence for the existence of life in old (e.g., the Precambrian) geological strata [142]. However, degraded microbial fossils often resemble one another in shape and size, and morphological taxonomy is usually not enough to determine the precise phylogenetic positions. High spatial resolution and non-destructive analyses make micro-FTIR particularly useful in distinguishing the phylogenetic position of microbial fossils. Igisu *et al.* [142] applied micro-FTIR to well-preserved microfossils from ~850 Ma and ~1900 Ma stromatolites, and demonstrated that CH_3_/CH_2_ absorbance ratios were a good proxy for the chemical composition of precursor membrane lipids and a useful indicator to distinguish Archaea, Bacteria, and possibly Eucarya (Figure 11).

The research of Igisu *et al.* [145] focused on demonstrating the feasibility of micro-FTIR mapping in distinguishing microfossils morphologically. They mapped two morphotypes of phosphatized embryo-like microfossils, together with algal fossils from the Doushantuo group, China. Displaying the distribution of aliphatic and aromatic functional groups inside the embryo-like fossils, micro-FTIR maps differentiated microfossils that are morphologically within the same taxon (see figure 15 in Igisu *et al.* [145]). The chemical characterization of microfossils together with a morphological analysis may provide new biological information on embryo-like fossils.

Additionally, FTIR data provide insightful chemical criteria for plant fossil taxonomic classification when it is complemented by known morphologically-derived taxonomic criteria [146,147,148,149]. Zodrow *et al.* [147] investigated well-preserved cuticles isolated from Geinitz leaf compressions with FTIR. The similarities between spectra in the three cuticular morphotypes supported their classification into a single cordaite-leaf taxon. The use of statistical methods, like principle component analysis (PCA), may aid in fossil-leaf chemotaxonomy when paired with FTIR techniques. For instance, D’Angelo *et al.* [149] introduced a new strategy for studying Carboniferous compression foliage by coupling FTIR and PCA analysis in order to gain insight into the types and relative proportions of structural groups in pinnules of the same segment. The results displayed a non-uniformity in the fossilization of Carboniferous foliage within a plant fossil for a given geological setting, which suggests variable physicochemical potentials driving OM transformation. These findings demonstrate that FTIR-assisted chemotaxonomy can be a valuable tool to further distinguish between plant-fossil groups.

## 4. Conclusions

Micro-FTIR provides insightful data on the chemical structure at the molecular scale, and is a very promising technique for the characterization of microscopic heterogeneity of geological samples like coal and shale. The chemical varieties within macerals can be well-documented and imaged with this non-destructive technique. More importantly, micro-FTIR provides a suitable and a straightforward way of tracing OM transformation across maturation by mapping shale samples of varying maturities. In addition, FTIR provides a fast, convenient, and accurate quantitative approach to investigate shale compositions that is complementary to XRD.

The non-destructive character of micro-FTIR enables chemical investigations of geological samples that are micrometer in size, such as fluid and melt inclusions hosted in minerals, and microfossils not easy to classify with other methods. Moreover, the possibility of real-time monitoring of chemical evolution in response to thermal stress by micro-FTIR provides a new opportunity to study problems associated with chemical reaction rates under non-ambient conditions. Thermal treatment, dehydration processes, and many other geochemical processes can benefit from this convenient, *in situ* technique. In addition, FTIR data can provide insightful chemical information for the taxonomic classification of plant fossils (and likely other macrofossils).
